# Controlling the defects and transition layer in SiO_2_ films grown on 4*H*-SiC via direct plasma-assisted oxidation

**DOI:** 10.1038/srep34945

**Published:** 2016-10-10

**Authors:** Dae-Kyoung Kim, Kwang-Sik Jeong, Yu-Seon Kang, Hang-Kyu Kang, Sang W. Cho, Sang-Ok Kim, Dongchan Suh, Sunjung Kim, Mann-Ho Cho

**Affiliations:** 1 Institute of Physics and Applied Physics, Yonsei University, Seoul, 120-749 Korea; 2 Department of Physics, Yonsei University, Wonju 220-710, Korea; 3 Department of Biomedical Engineering, Seonam University, Namwon 55724, Korea; 4 Process Development Team, Semiconductor R&D Center, SAMSUNG, Hwaseong-si 18448, Korea

## Abstract

The structural stability and electrical performance of SiO_2_ grown on SiC via direct plasma-assisted oxidation were investigated. To investigate the changes in the electronic structure and electrical characteristics caused by the interfacial reaction between the SiO_2_ film (thickness *~*5 nm) and SiC, X-ray photoelectron spectroscopy (XPS), X-ray absorption spectroscopy (XAS), density functional theory (DFT) calculations, and electrical measurements were performed. The SiO_2_ films grown via direct plasma-assisted oxidation at room temperature for 300s exhibited significantly decreased concentrations of silicon oxycarbides (SiO_*x*_C_*y*_) in the transition layer compared to that of conventionally grown (i.e., thermally grown) SiO_2_ films. Moreover, the plasma-assisted SiO_2_ films exhibited enhanced electrical characteristics, such as reduced frequency dispersion, hysteresis, and interface trap density (*D*_*it*_ ≈ 10^11^ cm^−2^ · eV^−1^). In particular, stress induced leakage current (SILC) characteristics showed that the generation of defect states can be dramatically suppressed in metal oxide semiconductor (MOS) structures with plasma-assisted oxide layer due to the formation of stable Si-O bonds and the reduced concentrations of SiO_*x*_C_*y*_ species defect states in the transition layer. That is, energetically stable interfacial states of high quality SiO_2_ on SiC can be obtained by the controlling the formation of SiO_*x*_C_*y*_ through the highly reactive direct plasma-assisted oxidation process.

Silicon carbide (SiC) possesses the potential for excellent performance in powered electronic device, with characteristics such as a wide bandgap (approximately 3.25 eV for 4*H*-SiC), high thermal conductivity (4.9 W cm^−1^K^−1^), and high electric breakdown field (2.5–3.5 MV/cm for SiC versus 0.6 MV/cm for Si), which make it suitable for high-power and high-frequency metal-oxide-semiconductor (MOS) devices with reduced power loss^1^,^2^,^3^,^4^,^5^,^6^,^7^. However, MOS field-effect transistors (MOSFETs) that are fabricated on 4*H*-SiC exhibit extremely low inversion-channel mobilities because of the high density of interface traps (*D*_*it*_) at the SiO_2_/4*H*-SiC interface^8^,^9^,^10^,^11^,^12^,^13^,^14^. According to recent studies, interface traps in SiC-based MOSFETs may be caused by the presence of excess C atoms, point defects, such as Si vacancies, and silicon oxycarbides (SiO_*x*_C_*y*_)^14^,^15^,^16^,^17^,^18^,^19^,^20^,^21^,^22^. The SiO_*x*_C_*y*_ species in the interfacial transition region (thickness ≈1 nm) are considered to be primarily responsible for the observed *D*_*it*_^18^. Therefore, a high-quality gate oxide (e.g., SiO_2_) with a well-engineered interface is essential for enhancing the performance of SiC-based MOSFET devices. In particular, reducing the concentrations of SiO_*x*_C_*y*_ species in the transition region of the SiO_2_ film is critical for making a high-quality gate dielectric. It has been reported that thermally grown SiO_2_ contains a transition layer that is less than 10 Å thick^23^. The transition region contains impurities (e.g., SiO_*x*_C_*y*_ species in the transition layer of the SiO_2_/SiC interface) and other defect sites, such as dangling bonds, strained bonds, interface traps, and fixed charges. These defects deteriorate the electrical properties of SiC-based MOSFET devices, such as the charge-to-breakdown, carrier mobility, and gate-leakage current. Furthermore, because the relative thickness of the transition region of the SiO_2_ film increases as the scale of such devices decreases, the fabrication of high-quality SiO_2_ gates on SiC is becoming more important.

Thermal oxidation is the standard method for growing high-quality SiO_2_ films. However, because the growth of SiO_2_ on SiC substrates in a furnace is time consuming (several minutes to hours) and requires high temperatures (800–1400 °C), a high thermal stress is inevitably induced during the oxidation process, which results in a thicker transition region (i.e., higher concentrations of SiO_*x*_C_*y*_ species) and the generation of defects (e.g., excess C atoms and O vacancies) at the SiO_2_/SiC interface^15^,^18^,^19^,^20^,^21^,^22^. Therefore, the interface between a SiO_2_ gate and SiC substrate is degraded during the thermal oxidation process by a greater extent than that of an equivalent SiO_2_ gate grown on a Si substrate. Recently, several studies have reported that the bulk traps caused by the generation of defects during high-temperature oxidation are responsible for the low mobility of 4*H*-SiC-based MOSFETs. Moreover, an excess of C atoms near the interface of the SiC substrate results in the formation of SiO_*x*_C_*y*_ species via a reaction process involving O_2_ and di-interstitial C clusters [(C_*i*_)_2_] that are formed through the pairing of interstitial C atoms, which is a major cause of the degraded performance of MOSFET devices with SiC substrates^24^,^25^,^26^,^27^,^28^.

In this study, the characteristics of SiO_2_ films grown on SiC substrates at room temperature for 300 s via direct plasma-assisted oxidation were investigated. A combination of experimental measurements and theoretical calculations showed that there is a clear difference between the formation of SiO_*x*_C_*y*_ species in the transition regions of thermally grown and plasma-assisted SiO_2_ films. The results of this study indicate that the plasma-assisted oxidation method is very effective in improving the electrical characteristics of SiO_2_/SiC systems because of the reduced concentrations of SiO_*x*_C_*y*_ species and the energetically stable interface states of such systems.

## Results and Discussion

**Figure 1:**
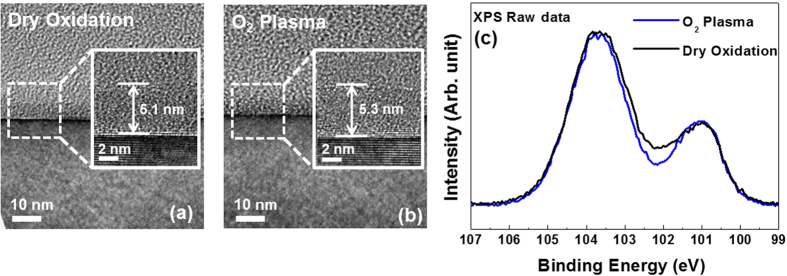
Cross-sectional HR-TEM images of the (**a**) thermally grown and (**b**) plasma-assisted SiO_2_ films on SiC substrates. (**c**) Si 2*p* core-level spectra of the thermally grown and plasma-assisted SiO_2_ films on SiC substrates.

Figure 1(a,b) show cross-sectional HR-TEM images of the thermally grown (dry oxidation) and plasma-assisted (O_2_ plasma oxidation) SiO_2_ films on SiC substrates, respectively. For both oxidation processes, SiO_2_ films with thicknesses of approximately 5 nm have been successfully grown via interfacial reactions on SiC substrates. To confirm the chemical states created by each oxidation process, XPS measurements (detection angle = 90°) were performed. The two main peaks in the Si 2*p* spectra of the SiO_2_ films (Fig. 1(c)) are caused by the fully oxidized state of the SiO_2_ films (Si^4+^ near 103.8 eV) and the bulk state of the SiC substrates (Si-C near 100.8 eV). However, there is a large difference in the chemical bonding states of the thermally grown and plasma-assisted SiO_2_ films, with the Si 2*p* spectrum of the thermally grown film containing an additional broad peak between 103 and 101.5 eV. This broad peak corresponds to the intermediate oxidation state of SiO_*x*_, which is indicative of unstable Si-O bonding near the SiO_2_/SiC transition region. Generally, the standard thermal oxidation process for growing SiO_2_ on Si-based semiconductors results in excellent electrical characteristics, such as a high intrinsic breakdown voltage and low leakage current. However, a side reaction occurs during the thermal oxidation of on SiC substrates, which results in the formation of SiO_*x*_C_*y*_ species or clusters of excess C atoms. That is, interstitial C, graphite-like clusters, and/or *sp*^2^-bonded clusters are formed at the interfacial transition region (thickness ≈ 1 nm) between the SiO_2_ film and SiC substrate, the presence of which negatively affects the reliability of SiC-based MOS devices^18^.

**Figure 2:**
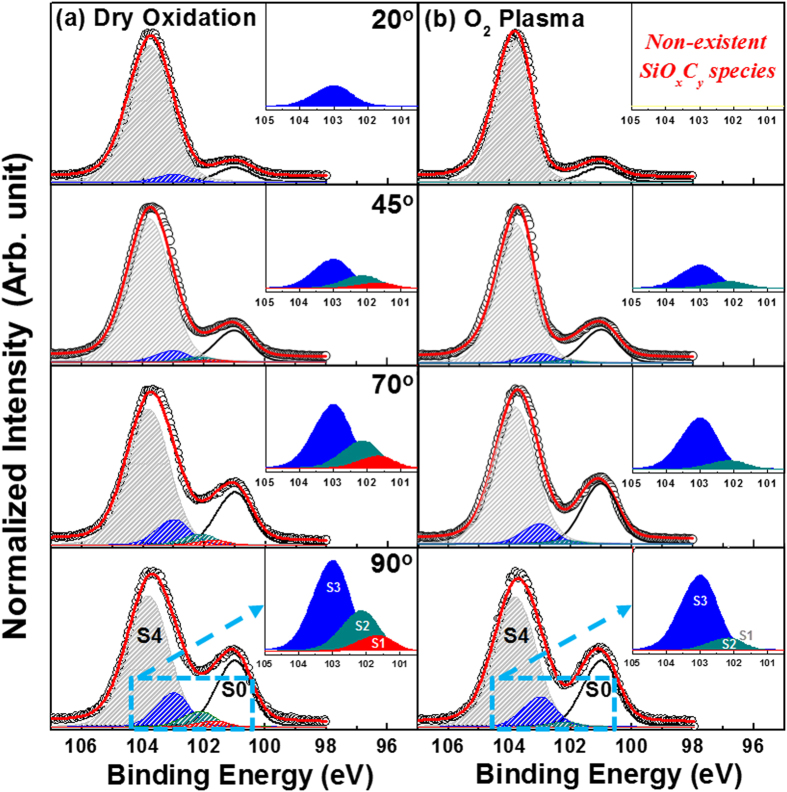
ADXPS spectra of the Si 2*p* core level as a function of detection angles (20^o^, 45^o^, 70^o^, 90^o^) for the (**a**) thermally grown and (**b**) plasma-assisted SiO_2_ films. The fitted spectra show that there is a significant reduction in the concentrations of intermediate SiO_*x*_C_*y*_ species in the plasma-assisted SiO_2_ film when compared to that of the thermally grown film.

**Figure 3:**
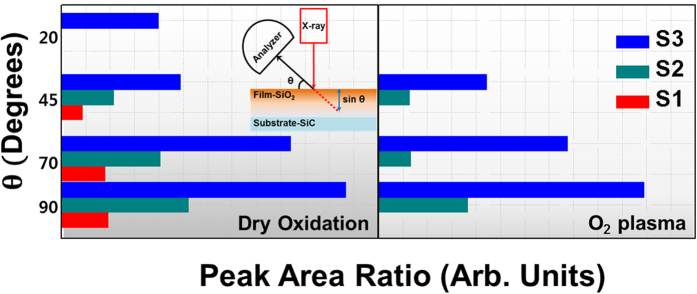
Quantitative depth distribution of the SiO_*x*_C_*y*_ species in the thermally grown and plasma-assisted SiO_2_ films. The changes in the chemical bonding ratios of S1, S2, and S3 were calculated from the peak areas of the ADXPS results.

The chemical bonding characteristics of the intermediate broad peak in the Si 2*p* spectra were investigated in detail because it is related to the SiO_*x*_C_*y*_ species in the transition region. The XPS spectra of the samples were measured at various detection angles to determine the distribution of SiO_*x*_C_*y*_ species along the depth direction of the thermally grown and plasma-assisted SiO_2_ films, as shown in Fig. 2. Recently, Zhu *et al.*^29^ measured the depth distributions of various SiO_*x*_C_*y*_ species near the SiO_2_/SiC interface as a function of polar emission angle via angle-dependent XPS (ADXPS). The ADXPS measurements showed that there is not an abrupt change between the chemical bonding states of the resulting SiO_2_ layer and the SiC substrate at the interface. That is, there is a transition layer with a thickness of approximately 1 nm at the interface, which Zhu *et al.* described as a four-layer structure. Zhu *et al.* reported that the first layer of the transition layer contained SiOC_3_, which is formed through the reaction of O with the sub-surface Si atoms of the SiC substrate. Moreover, the results of the ADXPS measurements showed that the second, third, and fourth layers of the transition layer had different depth distributions of SiO_2_C_2_ and SiO_3_C species. Prior to performing the curve fitting of the XPS results, a Shirley background subtraction was performed and all spectra were charge compensated relative to the binding energy of the Si-C bond (100.8 eV). The Si 2*p* spectra were decomposed into contributions from SiC, SiOC_3_, SiO_2_C_2_, SiO_3_C, and SiO_2_ species, which will hereinafter be referred to as S0, S1, S2, S3, and S4, respectively. The chemical shifts of the S1–S4 peaks relative to the S0 peak at 100.8 eV are 0.7, 1.2, 2.0, and 3.0 eV, respectively. The results of ADXPS curve fitting show that the concentrations of the S1–S3 species in the plasma-assisted SiO_2_ film decrease towards the subsurface of the film (i.e., traveling from the bulk SiC substrate to the SiO_2_ surface), which is unlike the results obtained in case of the thermally grown SiO_2_ film. In addition, in the plasma-assisted SiO_2_ film, the S1–S3 species are not detected when the detection angle = 20°. These changes in the concentrations of the S1–S3 species indicate that the formation of the S1–S3 species in the transition region is closely related to the different oxidation processes caused by using an O_2_ plasma process. For a detailed and quantitative analysis of the depth distributions of the SiO_*x*_C_*y*_ species, changes in the chemical bonding ratios of the S1–S3 species were compared by calculating the peak areas of the ADXPS results, as shown in Fig. 3. The peak areas corresponding to the S1 (non-existent) and S2 species in the plasma-assisted SiO_2_ film dramatically decrease in the film surface direction compared to that of the thermally grown film, with the SiO_*x*_C_*y*_ species no longer detected when the detection angle = 20°. The surface of the SiC substrate (atomic ratio of Si:C = 1:1) is gradually transformed into SiO_2_ (atomic ratio of Si:O = 1:2) during the oxidation process according to the reaction shown in Equation (1), which can be followed by a high-temperature oxidation process (Equation (2)). The reaction shown in Equation (2) involves dissociated C atoms that are created by the reaction shown in Equation (1), which are then incorporated into the SiO_2_ film at the interfacial region^19^.


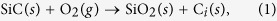



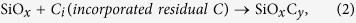


**Figure 4:**
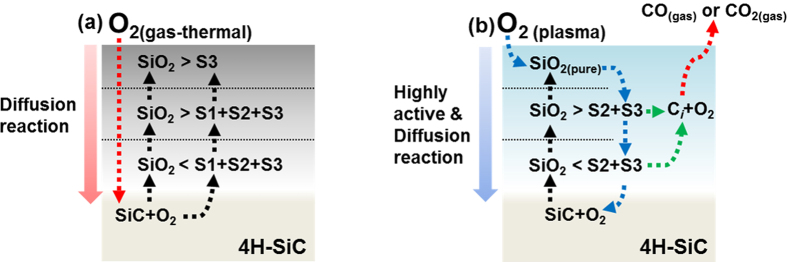
Schematic diagram of the reaction mechanisms involved in the (**a**) thermal and (**b**) plasma-assisted oxidation processes.

From the quantitative analysis of the depth distribution of SiO_*x*_C_*y*_ species in Fig. 3, a simplified reaction mechanism for each oxidation process was developed by considering the different diffusivities of the sources of O and the decomposition of Si and C at the SiO_2_/SiC interface during the initial growth stage, as shown in Fig. 4. The diffusion of O_2_ gas is not extremely fast during the thermal oxidation process. The O_2_ gas that inter-diffuses through the SiO_2_ film reacts with the SiC substrate at the SiO_2_/SiC interface, which results in the formation of mixed SiO_*x*_ states and the incorporation of C to create SiO_*x*_C_*y*_ species (S1, S2, and S3) at the transition region. On the other hand, during the plasma-assisted oxidation process, the highly activated and ionic O plasma, which is in either an atomic state or a radical state, can easily react with Si and C. The energy of the plasma is sufficient to form the more stable SiO_2_, which suppresses the incorporation of C into SiO_*x*_C_*y*_ species (S2 and S3). Moreover, the irradiation from the plasma-assisted oxidation process can break the unstable Si-O-C network structure, and the decomposed Si and Si-O can then react with the plasma to form the more stable SiO_2_^30^. The highly active O plasma can also react with defect sites in the SiO_2_ layer, i.e., it can easily react with unstable SiO_*x*_C_*y*_ species, resulting in the formation of the more stable SiO_2_. Simultaneously, C-based by-products, such as CO or CO_2_ (Equation (3)), are vaporized during the plasma-assisted oxidation process through reactions with decomposed C_*i*_ and C-O.


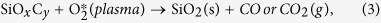


During the plasma-assisted oxidation process, some of the highly active O (ionic O) diffuses into the SiO_2_/SiC interface, while in the thermal oxidation process, it is mostly O_2_ that diffuses into the interface. Therefore, the thermal and plasma-assisted oxidation processes are very different, i.e., the former is controlled by interfacial reactions, while the latter is dependent upon the inter-diffusion process at the interface. In addition, the calculated formation energy (*ΔE*) for CO_2_ is extremely negative (Table 1; *ΔE* ≈ −21.939 eV, gas phase) in the 4*H*-SiC system. That is, it is easier to form CO_2_ during the plasma-assisted oxidation process than during the thermal oxidation process because of the reaction of the plasma with interstitial C atoms in the SiO_2_ film and SiO_2_/SiC interfacial region. The resulting CO_2_ then diffuses out as a gas through the thin SiO_2_ layer, and thus, it cannot contribute to the formation of SiO_*x*_C_*y*_ species. Moreover, the generation of a gas phase and its outwards diffusion can enhance the oxidation of Si because the interstitial C atoms that are removed by the reaction do not hinder the oxidation process at the SiO_2_/SiC interface. Based on the aforementioned reactions, the decrease in the S1–S3 peak areas towards the surface of the SiO_2_ film in the ADXPS data for the plasma-assisted sample imply that the formation of SiO_*x*_C_*y*_ species is effectively suppressed by the removal of C atoms.

**Figure 5:**
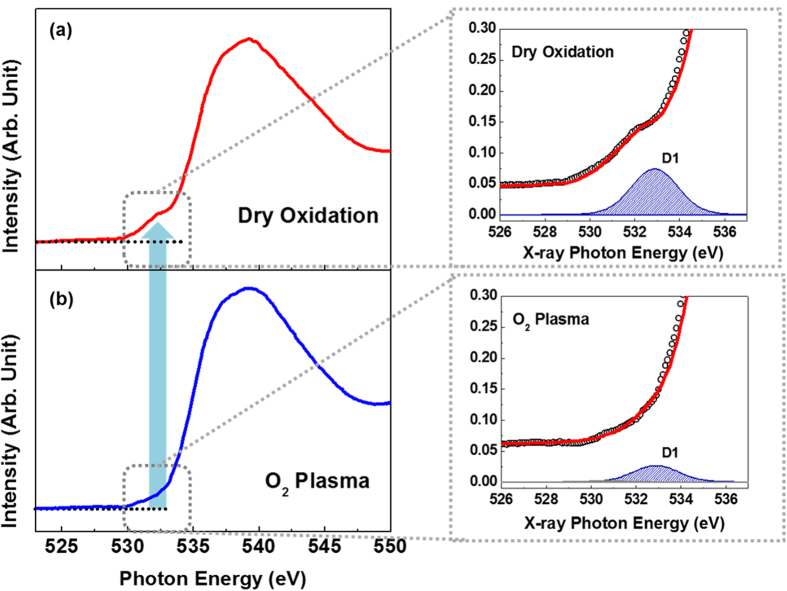
Normalized XAS O *K*-edge spectra of the (**a**) thermally grown and (**b**) plasma-assisted SiO_2_ films. The deconvoluted peak labeled D1, which indicate the presence of defect states, was obtained by applying Gaussian fits to the XAS O *K*-edge spectra.

To confirm the formation of stable Si-O bonds depending on the oxidation process, O *K*-edge XAS measurements were performed. The layer of SiO_*x*_C_*y*_ species in the transition region can induce defect states and disturb formation of stable SiO_2_ in the insulating bandgap. Figure 5 shows the XAS spectra of the thermally grown and plasma-assisted SiO_2_ films, which directly reflect the molecular orbital hybridization between the Si and O states based on the unoccupied *p*-projected DOS of O, with the local atomic bonding symmetry determined by dipole selection rules. The XAS results in Fig. 5(a,b) show an increase in the number of pre-edge states (indicated with a blue arrow). To analyze the absorption spectra in a narrow energy region of the pre-edge states, the renormalized O *K*-edge spectra were fitted with Gaussian peaks under the detection limit of the XAS set-up. The background was subtracted from the raw data via a straight background line before the normalization was performed. The difference in the pre-edge states is useful for comparing the relative magnitudes of the defect states in the O *K*-edge spectra. The energy states located near the pre-edge are evident after fitting the XAS data with Gaussian functions: peak D1 at 533 ± 0.2 eV. The D1 state is closely related to the unoccupied O states of SiO_*x*_, which can be defect states that induce gate-oxide leakage because of the O vacancies^23^. The D1 state was present in the thermally grown sample, while a reduced-intensity D1 state is exhibited by the plasma-assisted sample. The different intensities of the D1 states clearly indicate that the plasma-assisted oxidation process suppresses the generation of O vacancies and forms more stable SiO_2_, it is relatively reduction of formation of SiO_*x*_C_*y*_ species. This reduction in the number of defect states can significantly affect the characteristics of SiC-based MOSFET devices because the carrier scattering that is caused by defect states critically degrades device performance. Therefore, it is important to obtain high-quality SiO_2_ films on SiC substrates with suppressed interfacial defects for reliable device characteristics.

**Figure 6:**
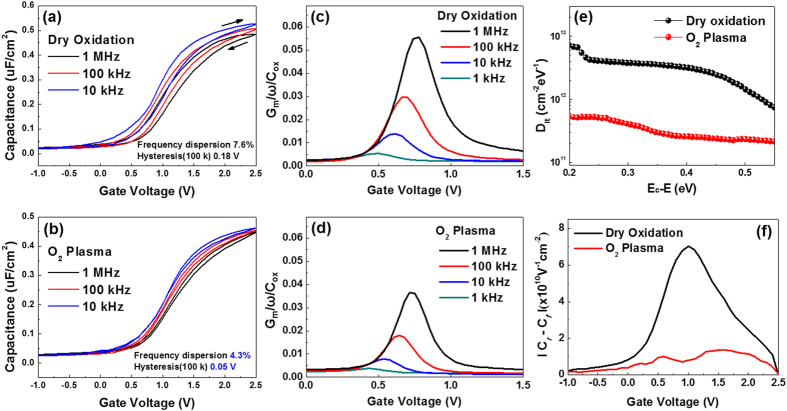
*C*-*V* curves of the (**a**) thermally grown and (**b**) plasma-assisted MOSCAPs when *f* = 1 MHz, 100 kHz, and 10 kHz and *G*-*V* curves of the (**c**) thermally grown and (**d**) plasma-assisted SiO_2_ films at each frequency (1 MHz, 100 kHz, 10 kHz and 1 kHz.). (**e**) *D*_*it*_ results for the thermally grown and plasma-assisted SiO_2_ films on SiC substrates at 25 °C. Effective border trap density at 100 kHz of (f) thermally grown and plasma-assisted SiO_2_ films.

To clarify the effects of plasma-assisted oxidation on the SiC substrate, electrical measurements were performed on a MOSCAP. Figure 6(a,b) show the *C*-*V* characteristics of the 5-nm-thick SiO_2_ films grown on SiC substrates via thermal and plasma-assisted oxidation methods, respectively, at three different frequencies. The most distinctive changes are the decrease in hysteresis at 100 kHz and the frequency dispersion of the plasma-assisted SiO_2_ film (approximately 0.05 V and 4.3%) compared to that of the thermally grown film (approximately 0.18 V and 7.6%). This change in the frequency dispersion is accompanied by a frequency-dependent flat-band shift, which is related to the weak Fermi-level pinning by the interfacial states rather than the series resistance^31^. Moreover, because the decrease in the *C*-*V* hysteresis is related to the reduction of the defect trap charge (O vacancies in the SiO_2_ film) in the plasma-assisted SiO_2_ film, the reduced frequency dispersion of the plasma-assisted sample can be interpreted as a decrease in the number of interfacial defect states. In addition, the *G-V* characteristics was evaluated using the conductance *G*_*m*_ normalized by the angular frequency (ω) versus the applied gate voltage (*V*_*g*_) of the both MIS structures (thermal and plasma oxidation), as shown in Fig. 6(c,d). The conductance peak values of plasma-assisted oxidation are lower than those for thermal oxidation at each frequency (1 M, 100 k, 10 k and 1 kHz). This indicates that the decreased conductance peak values of plasma oxidation are caused by the drastically low concentrations of interface traps and reduced surface potential fluctuations related to the charge exchange at the SiO_2_/SiC interface compared with interface traps of thermal oxidation. This is because of the advantages of the plasma-assisted oxidation process, such as the low processing temperature, highly activated oxidant, and short processing time. To confirm the differences between the interfacial defect states of the thermally grown and plasma-assisted SiO_2_/SiC systems, *D*_*it*_ values (*E*_*c*_ − *E*; 0.2–0.55 eV at room temperature) were determined via the typical conductance method. Figure 6(e) shows that the *D*_*it*_ value of the plasma-assisted SiO_2_ film is approximately 10^11^ cm^−2^·eV^−1^, which is much lower than that of the thermally grown film (*D*_*it*_ ≈ 10^12^ cm^−2^·eV^−1^). This result corresponds well with the aforementioned reduction in the frequency dispersion. The significant reduction of *D*_*it*_ in the case of the plasma-assisted oxidation process strongly reflects the control of the interfacial defect states at the SiO_2_/SiC interface. In addition to the *D*_*it*_ characterization, the differences between the border trap densities of the samples were investigated by measuring the *C-V* hysteresis characteristics at 100 kHz, as shown in Fig. 6(f). The shift in the *C-V* curve toward a positive voltage during the reverse sweep indicates that the electron trapping process communicates electrically via a slow response time, such that substrate injection into the SiO_2_ film occurs near the interface between SiO_2_/SiC, which is referred to as a border traps^32^. The border traps exchange the charges with the substrate on the time scale of the measurement. The *C-V* measurement was performed at a sweep rate as low as ~0.1 Vs^−1^ to encompass most of the border traps^33^. The effective border trap density per unit energy was calculated from the difference between the capacitances of the forward and reverse *C-V* scans [*C*_*rf*_* (V*_*g*_) = *|C*_*r*_ − *C*_*f*_ *|*], where *C*_*r*_ and *C*_*f*_ are the capacitance densities at a given *V*_*g*_ during the reverse and forward scans, respectively. The results show that the number of the effective border trap in the plasma oxidation sample (1.36 × 10^10^ V^−1^cm^−2^) is smaller than in the thermal oxidation sample (7.02 × 10^10^ V^−1^cm^−2^). It is known that slow traps can be located at the interfacial region between the oxide (thermal or plasma-SiO_2_) and the interfacial transition layer near the SiC substrate. Therefore, the high border trap density in the thermal-SiO_2_ sample could result from formation of oxygen vacancies and SiO_*x*_C_*y*_ species at the interface region during the thermal oxidation process, as observed in the previous XPS and XAS results. These defects influence the charge trapping and detrapping process, resulting in a large hysteresis. Finally, we can determine from the border trap characteristics that defect generation in the SiO_2_ oxide is more suppressed in the plasma-assisted oxidation process than in the thermal oxidation process. Moreover, we evaluated the electrical characteristics of the thermal and plasma oxidation samples using the thicker films (~30 nm SiO_2_). The frequency dispersion, hysteresis, *D*_*it*_, and border trap density are significantly decreased in the plasma-assisted oxidation sample when compared to the thermal oxidation sample, as shown in Fig. S2 of the supporting information. Therefore, the electrical results from the thin (~5 nm) and thick (~30 nm) films suggest that the plasma process used to obtain an energetic oxygen source is a successful approach to the growth of high-quality insulating films on SiC substrates for FET devices.

**Figure 7:**
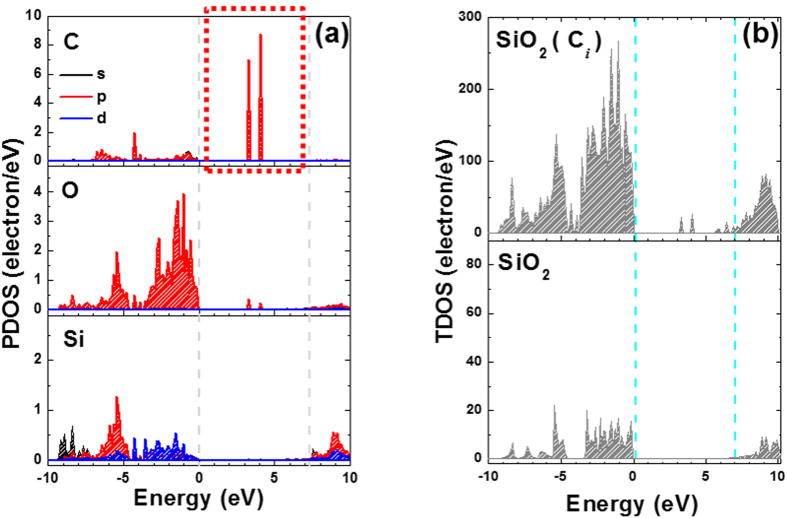
(**a**) PDOS calculations for the C, O, and Si atoms in SiO_2_ with a single interstitial C atom. (**b**) TDOS calculations for SiO_2_ with and without (pure) an interstitial C atom.

On the other hand, based on the electrical characteristics, the defects (high border traps and *D*_*it*_) are responsible for the degraded performance of 4*H*-SiC MOSFETs. Thus, to confirm the effects of the plasma-assisted oxidation process on the defective excess of C atoms, DFT calculations were performed to evaluate the interstitial C defect states in the SiO_2_ films. Using a unit cell with a single C atom in the interstitial site of the SiO_2_ structure, the partial density of states (PDOS) for the C, O, and Si atoms in the SiO_2_ film was evaluated by considering the optimized values for the atomic structure, as shown in Fig. 7(a). By comparing the PDOS of the C, O, and Si atoms at the interstitial C site of the SiO_2_ structure, it can be seen that the defect states within the SiO_2_ bandgap are predominantly generated by interstitial C atom (dashed red line). The PDOS results indicate that the electrical characteristics (higher frequency dispersion (*C*-*V*) and *D*_*it*_) of the thermally grown SiO_2_ film are significantly affected by the presence of interstitial C atoms at the SiO_2_/SiC interface. The corresponding changes in the total density of states (TDOS) of the SiO_2_ structure depending on the presence of interstitial C atoms were also confirmed, as shown in Fig. 7(b). The TDOS results show that the generation of defect states within the bandgap is controlled by the presence of interstitial C atoms within the SiO_2_ film. That is, the low border traps and *D*_*it*_ values of the plasma-assisted SiO_2_ clearly support the conclusion that the improvements in the electrical characteristics can be attributed to the reactions of the energetic plasma with the SiC substrate.

**Figure 8:**
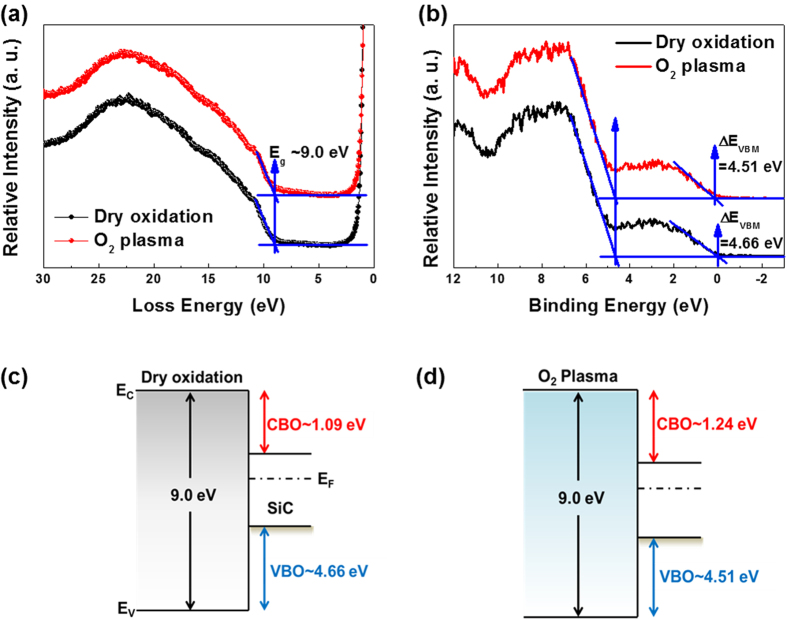
(**a**) REELS and (**b**) VB spectra of the thermally grown and plasma-assisted SiO_2_ films on SiC substrates. Schematic band diagrams for the (**c**) thermally grown and (**d**) plasma-assisted films, respectively.

To confirm the effects of the band structure (i.e., the conduction-band offset (CBO) and valence-band offset (VBO)) on the conduction mechanism, the REELS and valence band (VB) spectra of the thermally grown and plasma-assisted SiO_2_/SiC systems were obtained, as shown in Fig. 8. Energy-band parameters such as the bandgap (*E*_*g*_) can be defined as the threshold energy required for band-to-band excitation, as shown in the REELS spectra (Fig. 8(a)). The measured optical *E*_*g*_ of each SiO_2_ film is 9.0 ± 0.2 eV, which is almost the same as the literature value. To measure the VBO between the SiO_2_ film and SiC substrate, VB spectra were obtained for the thermally grown and plasma-assisted SiO_2_/SiC samples, as shown in Fig. 8(b). The effective electron-barrier height, i.e., the CBO, is calculated with Equation (4):


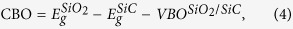


The energy-band diagrams of the thermally grown and plasma-assisted SiO_2_ films are shown in Fig. 8(c,d). In the plasma-assisted system, the effective hole-barrier height decreases to approximately 0.15 eV, while the effective electron-barrier height increases to approximately 0.15 eV.

**Figure 9:**
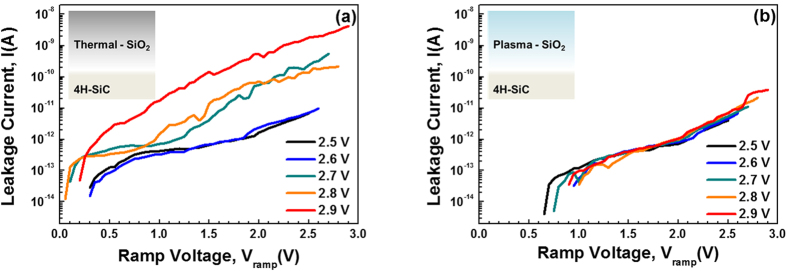
Leakage current as a function of the ramping voltage (SILC characteristics) for the (**a**) thermally grown and (**b**) plasma-assisted SiO_2_ films on SiC substrates.

Finally, the stability of the SiO_2_ films under voltage stressing conditions was evaluated by measuring the stress-induced leakage current (SILC). The *I*-*V* measurements were performed under a positive gate voltage (substrate electron injection). Successive forward *I*-*V* measurements with different ramping voltages were applied under ramped voltage stressing (RVS), as shown in Fig. 9. Once the stressing voltage increases beyond 2.6 V, the degree of leakage current in the *I*-*V* curve of the thermally grown SiO_2_ film increases. Since tunneling processes such as quantum mechanical tunneling and trap-assisted tunneling through the dielectric are increased by the electrical stress, defects (i.e., new electron traps) can be generated in the thermally grown SiO_2_ film by repeated high-field stresses. Thus, applying a high-field stress to the thin gate dielectric produces a leakage current. Hence, the increase in the leakage current of the thermally grown SiO_2_ film indicates that defects are generated in the film after a stressing voltage of 2.6 V is reached, which is lower than the breakdown voltage (3.0 V), as shown Fig. 9(a). On the other hand, with the plasma-assisted SiO_2_ film, the leakage current characteristics are unchanged within the breakdown voltage of 3.0 V, as shown in Fig. 9(b). In addition, we performed cumulative distribution measurements of XPS (detection angle = 90^o^) and SILC (stressing voltage of 2.9 V) for sufficient statistical analysis of reproduced samples using the direct plasma-assisted oxidation process under the same process condition (SiO_2_ thickness ~5 nm), as shown in Fig. S3. The results of quantitative quantity of the S1, S2 and S3 (SiO_*x*_C_*y*_ species) were suppressed in plasma oxidation samples and maintained their ratios. Moreover, the cumulated SILC measurements of the leakage current characteristics is unchanged below the hard breakdown voltage of 3.0 V at the stressing voltage of 2.9 V. Thus, direct plasma-assisted oxidation strongly suggests the suppressed formation of SiO_*x*_C_*y*_ and improved interfacial quality of the SiO_2_ film. The main reasons for the low leakage current under high applied fields are as follows. First, the CBO of the plasma-assisted system compared to that of the thermally grown system is relatively high; the CBO is the potential barrier for electrons, and it is determined from the energy-band diagram shown in Fig. 8(c). Second, the intrinsic defects, such as O vacancies and SiO_*x*_C_*y*_ species, in the interfacial transition layer are effectively suppressed in the plasma-assisted system. Thus, the difference between the SILCs of the thermally grown and plasma-assisted systems indicates that the plasma-assisted oxidation process has distinct advantages, such as controlling the electron-barrier height and defect states.

## Conclusions

In summary, the different characteristics of the transition regions of thermally grown and plasma-assisted SiO_2_ films on 4*H*-SiC substrates were demonstrated through a combination of experimental measurements and theoretical calculations. The plasma-assisted SiO_2_ film grown at room temperature for 300 s had significantly decreased concentrations of SiO_*x*_C_*y*_ species in the transition region compared to that of the thermally grown film. The decreased concentrations of SiO_*x*_C_*y*_ species in the plasma-assisted SiO_2_ film improved its electrical characteristics (reduced frequency dispersion, hysteresis, and *D*_*it*_ ≈ 10^11^ cm^−2^ · eV^−1^) and SILC characteristics. The results of this study indicate that the plasma-assisted oxidation process improves the quality of SiO_2_ films by suppressing the formation of SiO_*x*_C_*y*_ species in the interfacial transition region and encouraging the formation of an energetically stable interfacial state between the SiO_2_ film and SiC substrate, which is achieved by effectively preventing the formation of interstitial C atoms at the SiO_2_/SiC interface. The results of this study also show that the C atoms generated during the oxidation process play a significant role in inducing interfacial defect states through the formation of SiO_*x*_C_*y*_ species. Therefore, the physical and electrical results have shown that using a plasma source to obtain energetic O is a successful approach for the growth of high-quality insulating films on SiC substrates. Furthermore, the results of this study provide important information for improving the reliability of SiO_2_/SiC-based MOSFET devices. Therefore, simple and low-cost thin-film SiO_2_ can be rapidly fabricated on SiC substrates via a direct plasma-assisted oxidation process at room temperature. Finally, the results of this study suggest that an active oxidation process is highly favorable for the fabrication of high-power FET devices.

## Methods

### Experiment

SiO_2_ films that were approximately 5 nm thick were grown via thermal and plasma-assisted oxidation methods on the (0001) Si face of epi-ready, n-type, 4*H*-SiC substrates (doping concentration ≈ 1 × 10^18^ cm^−3^). Before performing the oxidation processes, the SiC substrates were cleaned for 1 min in a dilute solution (approximately 1%) of hydrofluoric (HF) acid. The thermal oxidation process was performed in a furnace for 20 min under a dry O_2_ atmosphere at 1050 °C. The plasma-assisted SiO_2_ films were grown in a remote radio-frequency (RF) plasma chamber at room temperature for 300 s under a flow of O_2_ gas (1000 sccm; ignition gas = Ar, 20 s) and plasma power of 1100–1400 W (supporting Fig. S1). The thicknesses of the SiO_2_ films on the SiC substrates were investigated via high-resolution transmission electron microscopy (HR-TEM). The HR-TEM images were obtained using a field-emission gun at an acceleration voltage of 200 keV. The energy bandgaps of the SiO_2_ films were measured via reflection electron energy-loss spectroscopy (REELS) with a primary beam energy of 1.0 keV. The chemical bonding states and valence band of the SiO_2_ films grown on the 4*H*-SiC substrates were examined via high-resolution X-ray photoelectron spectroscopy (XPS); a monochromatic Al *Kα* X-ray source (*hν* = 1486.7 eV) with a pass energy of 23 eV was used with detection angles of 20–90°. The SiC substrates were electrically grounded to the electron analyzer to eliminate any charging effects. The binding energies of the measured core-level spectra and valence band were calibrated relative to the SiC substrate peak in the Si 2*p* spectrum (100.8 eV for the Si-C bond). The characteristics of the defect states in the SiO_2_ films were examined via X-ray absorption spectroscopy (XAS); a synchrotron X-ray source at the X1B beam line of the National Synchrotron Light Source (NSLS) was used for these measurements. XAS photon energies of 520–560 eV (energy step = 0.05 eV) were used to measure the O *K*_1_-edge absorption spectra of the SiO_2_ films by using the total electron yield mode.

To examine the effects of the plasma-assisted oxidation method on a MOS capacitor (MOSCAP), a TiN film (area = 6.4 × 10^−5^ cm^2^; thickness = 200 nm) was deposited on a SiO_2_/SiC substrate via a top electrode via a lift-off technique. To obtain an accurate understanding of the interfacial defects, forming gas annealing (i.e., a defect-curing process) was not performed. Capacitance-voltage (*C*-*V*) and leakage current density-electric field (*J*-*E*) measurements were performed with an Agilent E4980A LCR meter and an Agilent B1500A semiconductor device analyzer, respectively. To compare the *D*_*it*_ values of the thermally grown and plasma-assisted SiO_2_ films, the capacitance (*C*_*m*_) and conductance (*G*_*m*_) of the films were measured. The *D*_*it*_ values were determined via calculations by using a combination of forward bias capacitance-frequency (*C*-*f* ) and *G*-*f* measurements to obtain the parallel conductance (*G*_*p*_), while the energy levels of the defect states were determined from frequency measurements^31^,^34^. The *G*_*p*_/*ω* values were calculated using Equation (5):


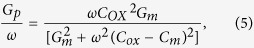


where *ω* = 2π*f*, *f* varies from 1 kHz to 1 MHz, and *C*_*ox*_ is the gate-oxide capacitance. A correction term was introduced to account for the leakage current, which was obtained from the current-voltage (*I*-*V*) curves. The *D*_*it*_ in the depletion region is proportional to the maximum value of *G*_*p*_/*ω*, as shown in Equation (6):


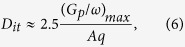


where *A* is the area of the electrode and *q* is the elemental charge. The trap energy level given by Shockley-Read-Hall (SRH) statistics for the carrier capture and emission rates was evaluated using Equation (7), which describes the relationship between the time constant of the trap (*τ*) and the frequency:


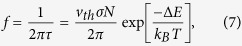


In addition, density functional theory (DFT) calculations were performed to understand the defect-formation energy of the C atoms (interstitial and converted atoms) in the SiO_2_ films by applying super cell models. The atomic structures and energy states were calculated with the Vienna Ab-initio simulation package; a generalized-gradient approximation functional, PBESol, was used for the exchange-correlation energy functional. First, geometry optimization was performed for the unit cell of the SiO_2_ structure. To evaluate the defect-formation energy and energy state, O sites or interstitial sites were replaced with C atoms and the geometry optimization was performed with a 3 × 3 × 1 super cell. DFT calculations for the geometry optimization and density of states (DOS) were then performed. Gamma k-points were used for the geometry optimization, and 3 × 3 × 1 k-points were used to calculate the energy state and DOS. All calculations were performed with a plane wave cut-off energy of 500 eV.

## Additional Information

**How to cite this article**: Kim, D.-K. *et al.* Controlling the defects and transition layer in SiO_2_ films grown on 4*H*-SiC via direct plasma-assisted oxidation. *Sci. Rep.* **6**, 34945; doi: 10.1038/srep34945 (2016).

## Supplementary Material

Supplementary Information

## Figures and Tables

**Table 1:** Formation energies of the 4*H*-SiC system for various structures and phases.

Structure	*ΔE* (eV)	Phase
Si	−5.290233	Solid
CO_2_	−21.939852	Gas
CO	−13.972246	Gas
C	−9.08395075	Solid

The *ΔE* values were obtained via DFT calculations.
