# Supplementary material for: Controlling the defects and transition layer in SiO2 films grown on 4H-SiC via direct plasma-assisted oxidation
Source: Sci Rep. 2016 Oct 10;6:34945. doi: 10.1038/srep34945 (PMC5056351; doi:10.1038/srep34945)
Supplement: Supplementary Information [file srep34945-s1.doc]

**Controlling the defects and transition layer in SiO_2_ films grown on 4*H*-SiC via direct plasma-assisted oxidation**

Dae-Kyoung Kim^1^, Jeong-Kwang Sik^1^, Yu-Seon Kang^1^, Hang-Kyu Kang^1^, Sang W. Cho^2^, Sang-Ok Kim^3^, Dongchan Suh^4^, Sunjung Kim^4^ and Mann-Ho Cho^1,*^

*^1^Institute of Physics and Applied Physics, Yonsei University, Seoul, 120-749 Korea*

*^2^Department of Physics, Yonsei University, Wonju 220-710, Korea*

*^3^Department of Biomedical Engineering, Seonam University, Namwon 55724, Korea*

*^4^ Process Development Team, Semiconductor R&D Center, SAMSUNG,* *Hwaseong-si 18448, Korea*

*Corresponding author mail address: [mh.cho@yonsei.ac.kr](mailto:mh.cho@yonsei.ac.kr) (Mann-Ho Cho).

**Supplementary information**


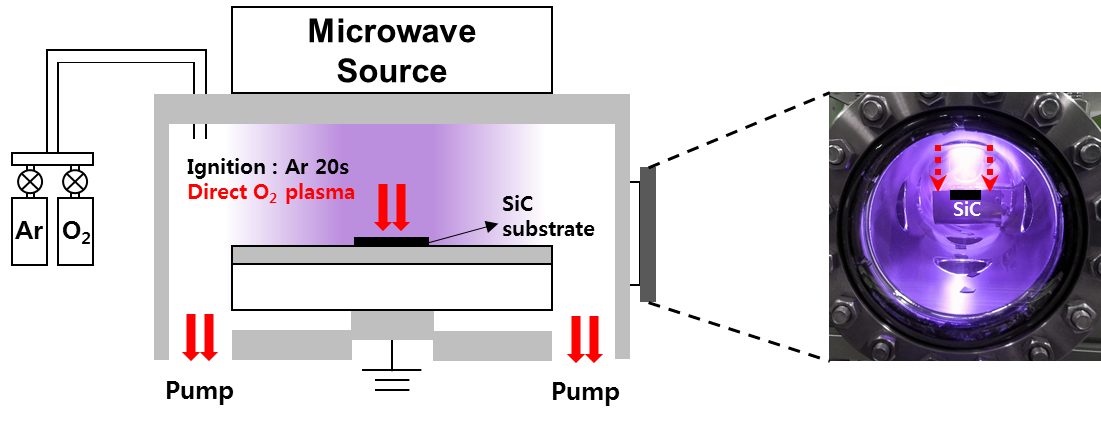


**Supplementary Fig. S1.** Schematic diagram of O_2_ Plasma oxidation process, oxidation was performed at room temperature for 300 s under a 1000 sccm O_2_ gas (ignition Ar, 20 s, working pressure 1 Torr) and plasma power 1100 – 1400 W.


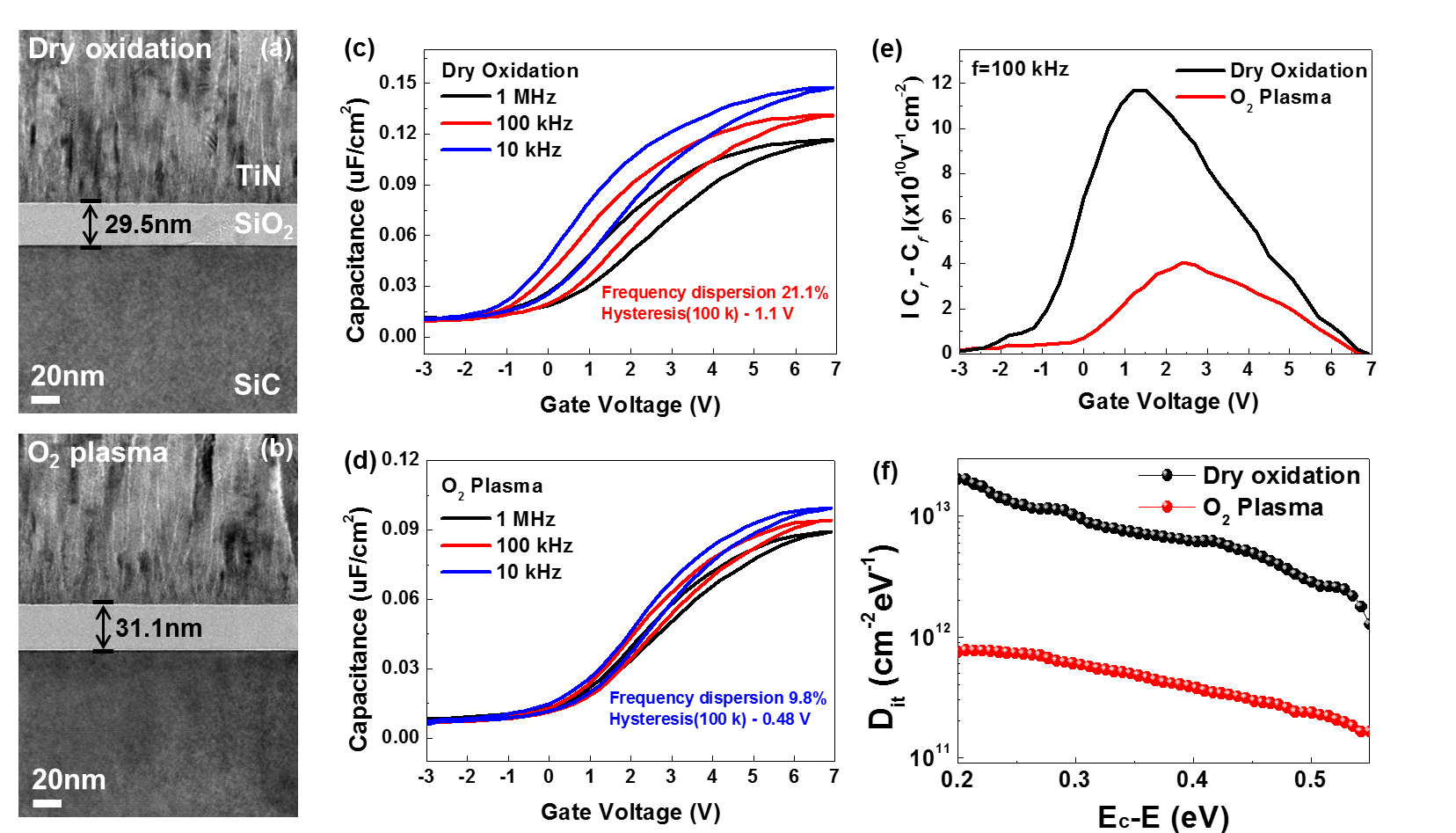


**Supplementary Fig. S2.** Cross-sectional HRTEM images of the (a) thermally grown and (b) plasma-assisted SiO_2_ (30 nm) on SiC and TiN metal gate deposition MOSCAPs. The *C-V* curves of the (c) thermally grown and (d) plasma-assisted SiO_2_. The Effective border trap density (e) at 100 kHz of *C-V* curves and (f) *D_it_* results of the thermally grown and plasma-assisted SiO_2_ films.


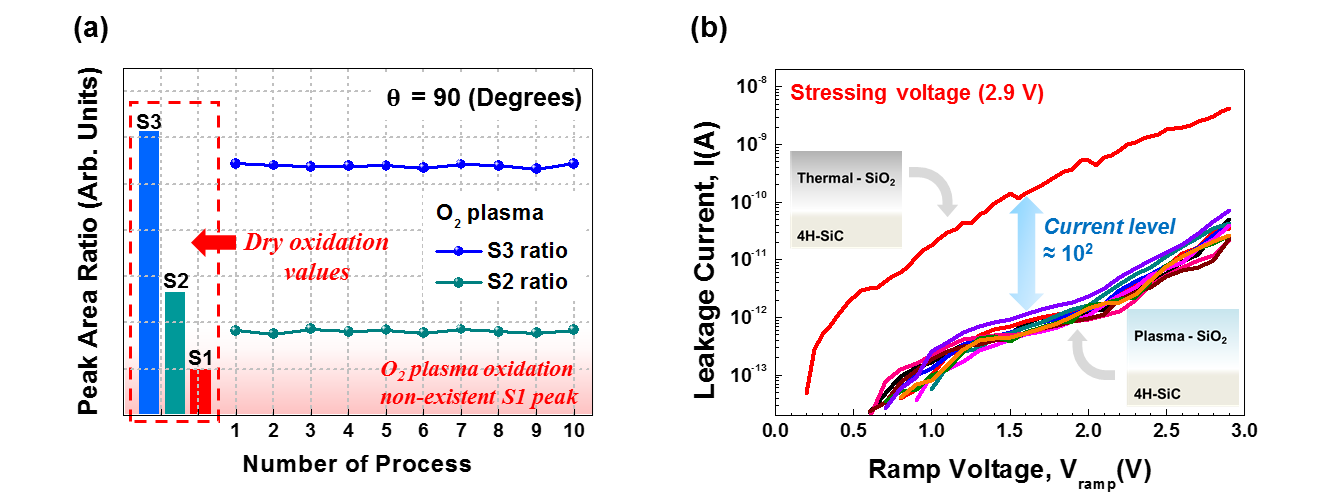


**Supplementary Fig. S3.** The quantitative distributions ratios (a) of the S1, S2 and S3 (SiO*_x_*C*_y_* species) in reproduced plasma oxidation samples and (b) cumulated SILC measurement of the leakage current characteristics of reproduced plasma oxidation samples at after stressing voltage 2.9 V.
